# Clinical outcomes and patterns of population‐based management of urachal carcinoma of the bladder: An analysis of the National Cancer Database

**DOI:** 10.1002/cam4.4786

**Published:** 2022-05-04

**Authors:** Furkan Dursun, Kelvin Lim, Robert S. Svatek, Jiaqiong Xu, Ziad M. El‐Zaatari, Evan P. Wenker, Zachary W. Klaassen, Ahmed M. Mansour, Taliah Muhammad, Eleni Efstathiou, Guru P. Sonpavde, Christopher J. D. Wallis, Raj Satkunasivam

**Affiliations:** ^1^ Department of Urology University of Texas Health San Antonio San Antonio Texas USA; ^2^ Department of Urology Houston Methodist Hospital Houston Texas USA; ^3^ Center for Outcomes Research Houston Methodist Hospital Houston Texas USA; ^4^ Department of Pathology and Genomic Medicine Houston Methodist Hospital Houston Texas USA; ^5^ Division of Urology Medical College of Georgia–Augusta University Augusta Georgia USA; ^6^ Department of Medical Oncology Houston Methodist Hospital Houston Texas USA; ^7^ Department of Genitourinary Medical Oncology Dana‐Farber Cancer Institute Boston Massachusetts USA; ^8^ Department of Urology University of Toronto Toronto Ontario Canada; ^9^ Department of Urology Mount Sinai Hospital Toronto Ontario Canada

**Keywords:** partial cystectomy, perioperative chemotherapy, survival outcome, radical cystectomy, urachal carcinoma

## Abstract

**Background:**

Given the low incidence of urachal carcinoma of the bladder (UCB), there is limited published data from contemporary population‐based cohorts. This study aimed to describe demographic, clinicopathological features, and survival outcomes of patients diagnosed with UCB.

**Methods:**

The National Cancer Database (2004–2016) was queried for UCB patients. Descriptive analyses characterized demographics and clinicopathologic features. We assessed 5‐year overall survival (OS) rates of the entire cohort and subgroups of localized/locally advanced and metastatic disease. We utilized Cox proportional hazards models to assess the association between covariates of interest and all‐cause mortality and to examine the impact of surgical technique and chemotherapy.

**Results:**

We identified 841 patients with UCB. The most common histologic subtype was non‐mucinous adenocarcinoma (39.6%). Approximately 50% had ≥cT2 disease, and 14.3% were metastatic at diagnosis. Altogether, partial cystectomy (60%) was most performed, and lymph node dissection was performed in 377 patients (44.8%), with specific temporal increase in utilization over the study period (*p* < 0.001). Overall, median OS was 59 months, and 5‐year OS was 49%. In patients with localized/locally advanced disease, we found no association between partial and radical cystectomy (Hazards ratio [HR] 1.75; 95% CI 0.72–4.3) as well as receipt of perioperative chemotherapy (HR 1.97, 95% CI 0.79–4.90) and outcomes. Lastly, receipt of systemic therapy was not associated with survival benefit (HR 0.785, 95% CI 0.37–1.65) in metastatic disease cohort.

**Conclusion:**

This large population‐based cohort provides insight into the surgical management and systemic therapy, without clear evidence on the association of chemotherapy and survival in the perioperative and metastatic setting.

## Graphical abstract

This large population‐based cohort of urachal carcinoma of the bladder (UCB) provides insight into the surgical management and systemic therapy, without clear evidence on the association of chemotherapy and survival in the peri‐operative and metastatic setting. Prospective, multi‐intuitional studies are needed to identify multi‐modal approaches to improve outcomes for patients with UCB.

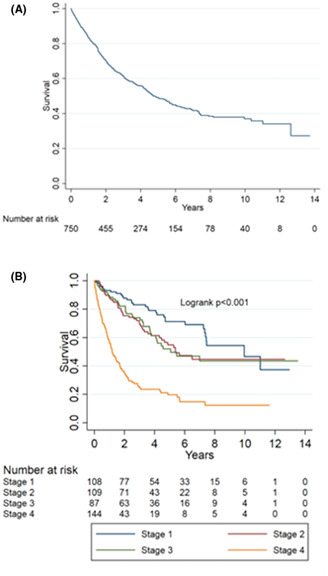

## 1 INTRODUCTION

The urachus usually regresses during fetal development to form the median umbilical ligament, but one‐third of the population fail to achieve complete obliteration. Though a persistent urachal remnant remains mainly benign, it can give rise to more sinister pathologies later in life, such as urachal cancer.^1^ Urachal carcinoma of the bladder (UCB) is a rare non‐urothelial neoplasm that accounts less than 1% of all bladder malignancies. The vast majority, comprising 85%–95%, of urachal carcinoma are histologically glandular and specifically adenocarcinoma, predominantly mucinous in nature, followed by non‐mucinous adenocarcinoma. Other glandular non‐adenocarcinoma (e.g., signet cell) or non‐glandular histology (e.g., urothelial or squamous cell carcinoma) are rare.^2^, ^3^, ^4^, ^5^, ^6^ The prognosis of UCB is poor, with patients presenting symptomatically with locally invasive or metastatic disease after an indolent clinical course, owing to the extravesical and extraperitoneal location of the urachus.^5^, ^7^

Due to the substantially low incidence of urachal cancer, the current diagnostic and treatment evidence is primarily derived from single‐institution series, and there is limited published data from contemporary population‐based cohorts.^8^ The standard treatment in localized disease involves surgical extirpation with either radical cystectomy (RC) or partial cystectomy (PC), in addition to en‐bloc resection of the umbilical ligament and umbilicus.^9^ Although systemic chemotherapy is the preferred treatment modality in patients with non‐localized or metastatic disease, the preferred regimen remains controversial.^10^ The role of neoadjuvant or adjuvant chemotherapy in patients receiving surgical resection remains unclear.

We sought to utilize population‐based data to inform contemporary patient and disease‐ related features of UCB, and patterns of treatment using the National Cancer Database (NCDB). We focused on two patient subgroups (localized/locally advanced and metastatic UCB) to describe clinicopathologic features and analyzed the association between survival outcomes and histologic types, varying treatment strategies. This included surgical treatments (PC or RC and the role of lymph node dissection [LND]) in localized/locally advanced disease, and the role of systemic chemotherapy in metastatic disease.

## 2 MATERIALS AND METHODS

### 2.1 Data sources

The NCDB is a joint project between the Commission on Cancer (CoC) of the American Cancer Society and American College of Surgeons. It is a contemporary, hospital‐based database that captures approximately 70% of new cancer diagnoses in the US annually, based on data collected from more than 1500 participating CoC accredited hospitals.^11^

### 2.2 Study population

The NCDB was queried to identify patients with International Classification of Disease for Oncology, Third Edition, (ICD‐O‐3) topographical code for urachal carcinoma (C67.7) who were aged 18 to 90 years old and diagnosed between 2004 and 2016. The staging information for patients was coded using the American Joint Committee on Cancer (AJCC) staging system for bladder cancer. For analytic purposes, patients with in‐situ and stage 1 disease were grouped together as stage 1. Patients with 1cT1–cT4, cN0–1, and cM0 were included in our entire cohort.

### 2.3 Covariates

Demographic and clinical data included age, sex, race, Charlson comorbidity index (CCI), residence (urban, metropolitan, and rural), and insurance status. Facility‐level variables included location (community centers, comprehensive community centers, and academic centers) and the degree of practice specialization. Disease‐related characteristics included tumor histology, clinical T‐stage, grade, presence of distant metastasis, regional lymph node positivity, tumor size, surgical approach, surgical margin status, and treatment modality. Site‐specific surgery codes were used to identify surgical procedures, as local tumor excision (10–16, 20–27), PC (30), and RC (60–64, 70–74, 80). Excision of metastatic lesions was assessed according to the Facility Oncology Registry Standards.^12^

### 2.4 Outcomes

The primary outcome was overall survival (OS) from the initial diagnosis to the date of death or last follow‐up.

### 2.5 Statistical analysis

Demographic, clinicopathologic, and treatment data of patients with diagnosis of urachal carcinoma are presented as median [interquartile range (IQR)] for continuous variables and number (percentage) for categorical variables. OS was estimated using the Kaplan–Meier analyses for staging subgroups, and the Log‐rank testing was used to determine significant OS differences.^13^ The survival at 5‐year intervals was compared with the pseudo‐value approach.^14^

Cox proportional hazards models were utilized to determine the association between survival and patient‐, facility‐, and disease factors. The global test, based on Schoenfeld residuals, showed that the proportional hazard assumption was violated for several variables, including histology, clinical staging, receipt of PC or RC, tumor size, and receipt of systemic therapy (neoadjuvant or adjuvant). Therefore, a multivariable Cox model was built, with an interaction term with time for violated variables along with other covariates. Hazard ratios were derived for 1, 3, and 5‐year time periods for interpretability.^15^

Patients were subdivided into two cohorts for subgroup analysis. The first consisted of localized and locally advanced urachal carcinoma (cT2–4, cN0–1, cM0) to evaluate the association of varying surgical management (PC or RC), nodal dissection, and perioperative chemotherapy and survival. Patients with ≤cT1 were excluded from this subgroup survival analysis because patients harboring this stage of disease do not typically undergo systemic therapy. A second cohort, consisting of patients with metastatic disease (Stage 4), was created to evaluate the association between chemotherapy and survival. The proportional hazard assumptions were met in both subgroups. Statistical significance was defined as two‐tailed *p* < 0.05 for all tests. All analyses were performed with STATA version 16 (Stata Corp.).

## 3 RESULTS

### 3.1 Demographic, clinicopathologic, and tumor characteristics

Baseline demographic, clinicopathologic, tumor characteristics are summarized in Table 1. Overall, a total of 841 patients with UCB were identified. The median age at diagnosis was 58 years (IQR 47–69), 57% male, and the predominant race were non‐Hispanic White (70%), followed by African American (12.6%). The majority of patients were privately insured (49.6%), lived in a metro area (81.2%), and received care in academic centers (77.3%), associated with a University Medical School or designated as Comprehensive Cancer Programs.

**TABLE 1:** Demographic, clinical, and histopathologic characteristics of patients with malignant urachal carcinoma (*n* = 841)

	Number (%)
Age	
≤50	262 (31.15)
51–60	202 (24.02)
61–70	197 (23.42)
71–80	118 (14.03)
>80	62 (7.37)
Sex	
Male	480 (57.07)
Female	361 (42.93)
Race	
White	588 (69.92)
Black	106 (12.6)
Hispanic	66 (7.85)
Asian	16 (1.9)
Other/unknown	65 (7.73)
Insurance status	
Not insured	38 (4.52)
Private	417 (49.58)
Governmental	363 (43.16)
Unknown	23 (2.73)
Area of residence	
Metro	658 (81.23)
Urban	131 (16.17)
Rural	21 (2.59)
Annual income	
Less than $40,227	184 (22.14)
$40,227–$50,353	171 (20.58)
$50,354–$63,332	175 (21.06)
$63,333+	301 (36.22)
Charlson/Deyo Score	
0	653 (77.65)
1	148 (17.60)
≥2	40 (4.76)
Follow‐up, median months (IQR)	31.6 (13.7–63.2)
Facility type	
Community Cancer Program	70 (9.35)
Comprehensive Community Cancer Program	274 (36.58)
Academic/Research Program	305 (40.72)72
Integrated Network Cancer Program	100 (13.35)
Clinical T‐stage	
≤T1	134 (20.95)
T2	97 (15.16)
T3	51 (7.97)
T4	9 (1.41)
Tx	349 (54.53)
Clinical N stage	
N0	525 (65.30)
N+	46 (5.72)
Nx	233 (29.0)
Clinical M stage	
M0	681 (85.45)
M1	114 (14.30)
Mx	2 (0.25)
AJCC overall stage	
Stage ≤1	127 (15.10)
Stage 2	124 (14.74)
Stage 3	92 (10.94)
Stage 4	169 (20.10)
Unknown/NA	329 (39.12)
Pathologic nodal status	
pN0	309 (36.74)
pN1/2	73 (8.68)
pNx	444 (52.79)
Missing	15 (1.78)
Surgical margins	
No residual tumor	526 (62.54)
Positive surgical margin	100 (11.90)
No primary site surgery	215 (25.56)
Histology	
Glandular	717 (85.26)
Non‐mucinous adenocarcinoma	333 (39.6)
Mucinous adenocarcinoma	329 (39.12)
Signet ring cell carcinoma	48 (5.71)
Other adenocarcinoma	7 (0.83)
Non‐glandular	124 (14.74)
Urothelial carcinoma	86 (10.23)
Squamous cell carcinoma	13 (1.55)
Other	25 (2.97)
Grade	
Well differentiated	111 (13.20)
Moderately differentiated	263 (31.27)
Poorly differentiated	191 (22.71)
Undifferentiated; anaplastic	56 (6.66)
Not determined, not stated or NA	220 (26.16)
Tumor size, mm, median (IQR)	45 (30–70)

Clinical T‐stage was ≤T1 in 21%, T2 in 15.2%, and ≥T3 in 9.4% at the time of diagnosis. The most common, known clinical nodal stage was cN0 in 525 patients (65.3%), followed by unknown or not examined nodal status in 233 (29.0%). Only 46 patients (5.7%) had clinically suspected nodal disease at diagnosis. Most patients were diagnosed with non‐metastatic disease (85.5%), and 14.3% of patients presented with suspected metastatic disease.

The majority of the tumors were histologically noted to be either moderately (31.3%) or poorly (29.4%) differentiated. Overall, urachal tumors predominantly harbored glandular histologic features (85.3%), with the most common histologic subtypes being non‐mucinous adenocarcinoma (39.6%) and mucinous adenocarcinoma (39.1%). When comparing all histological subtypes of UCB, signet ring cell (66.7%), urothelial (51.2%), and squamous cell carcinomas (46.2%) presented with worse histologic grade with poorly differentiated or undifferentiated Table S1. The median size of primary tumor at presentation was 45 mm (IQR 30–70).

### 3.2 Treatment patterns and temporal trends

The management of urachal cancer in the entire cohort was primarily by surgical resection (86.6%), of which PC was found to be the most common surgical procedure (60.4%), followed by local tumor excision (18%) and RC (8.4%) (Table 2). Systemic chemotherapy was utilized in 30.3% of patients overall (*n* = 256). Among patients who were treated with chemotherapy, those with locally advanced or metastatic disease were the most prevalent population (61.4%), as expected. The combination of surgical management with radiation was less common (*n* = 59, 7%), but when given, most cases were adjuvant radiation (*n* = 45, 76.3%). In the subgroup of patients with localized or locally advanced disease, the majority underwent PC (*n* = 118/139, 84.9%). Those undergoing PC were more likely to harbor glandular histology (*p* = 0.012) and undergo concurrent LND (*p* = 0.004) as compared to RC (Table S2).

**TABLE 2:** Treatment characteristics of patients diagnosed with urachal carcinoma

	Number (%)
Surgical procedure of the primary site	
No surgery of primary site	89 (10.58)
Local tumor destruction or excision	151 (17.95)
Partial cystectomy	508 (60.40)
Cystectomy	71 (8.44)
Missing	22 (2.62)
Regional lymph node surgery	
No	454 (53.98)
Yes	377 (44.83)
Missing	10 (1.19)
Chemotherapy at any facility	
Single agent	50 (5.95)
Multi agent	191 (22.71)
Single or Multi agent	15 (1.78)
No chemotherapy	573 (68.23)
Missing	12 (1.43)
Immunotherapy at any facility	
No	819 (97.39)
Yes	14 (1.66)
Missing	8 (0.95)
Systemic surgery sequence	*N* = 744
No systemic treatment or surgery	539 (64.10)
Systemic before	11 (1.30)
Systemic after	156 (18.55)
Other/unknown	38 (4.52)
Missing	97 (11.53)
Type of radiation	
None	771 (91.68)
Beam radiation	61 (7.25)
Other	9 (1.07)
Radiation surgery sequence	
None	782 (92.98)
Adjuvant radiotherapy	45 (5.35)
Other	14 (1.66)

At the time surgery, approximately 45% of patients underwent regional LND. When we examined the rate of LND between patients who underwent PC or RC by the year of surgery, we identified an increase over the study period for PC (43.5% in 2006 to 72.6% in 2014, Cochran‐Armitage test for trend *p* < 0.001); however, despite the increased utilization of LND at the time of RC from 50% in 2004 to 100% in 2016, the increased trend was not found to be statistically significant (Cochran‐Armitage test for trend *p* = 0.42), likely owing to the small number of patients undergoing RC.

### 3.3 Overall survival

**FIGURE 1:**
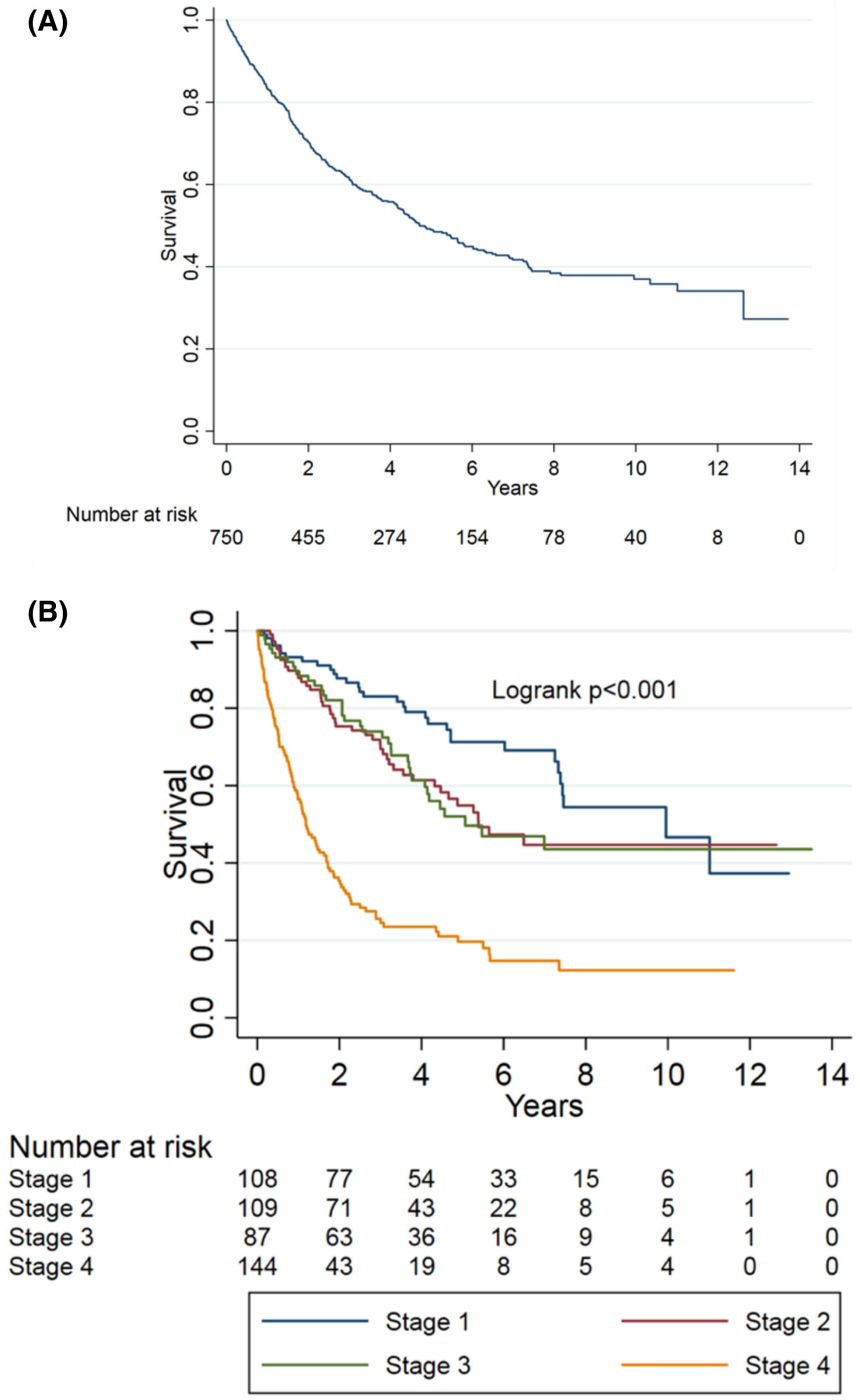
Kaplan–Meier survival curve. (A) Overall survival (OS) for the entire cohort of patients. (B) OS stratified by American Joint Committee on Cancer Clinical Stages; *p* < 0.001

Median follow‐up of patients with UCB, in whom survival data were available, was 31.6 months (IQR 13.7–63.2). Median OS for the entire cohort was 59 months, and the estimated 5‐year OS was 49% (95% CI 44.8–53) (Figure 1A). Using Kaplan–Meier analyses, there was a significant difference in OS among AJCC clinical stages (log‐rank *p* < 0.001; Figure 1B), with the following 5‐year OS rates: Stage I, 71.3%; Stage II, 54.9%; Stage III, 52.0%; and Stage IV, 19.7%.

Multivariable Cox proportional hazards model demonstrated several predictors of OS (Table 3). We modeled the association between OS and covariates of interest with time as an interaction term to account for violation of the proportional hazard assumption. As expected, age (*p* = 0.003), stage (*p* < 0.001), tumor size (*p* = 0.02), and either PC or RC (*p* = 0.002 and *p* < 0.001, respectively) were associated with OS. Most notably, signet ring cell carcinoma, a non‐adenocarcinoma histology, was associated with an increased risk of all‐cause mortality (HR 6.02; 95% CI 3.56–10.16; *p* < 0.001). Receipt of multi‐agent chemotherapy was also associated with higher risk of all‐cause mortality (HR 1.71; 95% CI 1.20–2.43; *p* = 0.003) compared to no chemotherapy. However, tumor grade and CCI were not independently associated with worse survival than well‐ or moderately differentiated tumor and CCI of 0.

**TABLE 3:** Multivariable extension of Cox Proportional Hazards Model with Time Interaction model to examine the association between demographic, clinical, and tumor variables, and overall survival for the entire cohort (*n* = 841)^a^

	Cox proportional hazards model with time interaction*	
	HR (95% CI)	*p*‐value
Age	1.01 (1.00–1.02)	0.003*
Charlson/Deyo score		
0	Referent	
1	1.19 (0.89–1.6)	0.242
2 and 3	1.37 (0.84–2.22)	0.205
Tumor grade		
Well and moderately	Referent	
Poorly and undifferentiated	1.23 (0.93–1.64)	0.147
Histology		
Non‐mucinous adenocarcinoma	Referent	
Mucinous adenocarcinoma		
At 1 year	2.77 (2.03–3.78)	<0.001*
At 3 year	1.21 (0.93–1.58)	0.15
At 5 year	0.53 (0.39–0.72)	<0.001*
Signet ring cell carcinoma		
At 1 year	6.02 (3.56–10.16)	<0.001*
At 3 year	2.63 (1.65–4.20)	<0.001*
At 5 year	1.15 (0.72–1.84)	0.55
Clinical T‐stage		
Stage 1 and 2	Referent	
Stage 3		
At 1 year	2.31 (1.45–3.70)	<0.001*
At 3 year	1.44 (0.93–2.33)	0.11
At 5 year	0.89 (0.58–1.39)	0.62
Stage 4		
At 1 year	7.08 (3.95–12.67)	<0.001*
At 3 year	4.40 (2.58–7.52)	<0.001*
At 5 year	2.74 (1.65–4.56)	<0.001*
Cancer‐related surgery		
No	Referent	
Local		
At 1 year	1.16 (0.75–1.78)	0.51
At 3 year	0.53 (0.35–0.80)	0.002*
At 5 year	0.24 (0.15–0.38)	<0.001*
Partial cystectomy		
At 1 year	2.26 (1.35–3.80)	0.002*
At 3 year	1.03 (0.67–1.60)	0.88
At 5 year	0.47 (0.32–0.71)	<0.001*
Radical cystectomy		
At 1 year	6.91 (3.75–12.72)	<0.001*
At 3 year	3.16 (1.87–5.33)	<0.001*
At 5 year	1.44 (0.90–2.32)	0.13
Tumor size		
≤45 mm	Referent	
>45 mm		
At 1 year	1.45 (1.06–1.99)	0.022*
At 3 year	0.93 (0.70–1.23)	0.60
At 5 year	0.59 (0.42–0.83)	0.002*
Receipt of systemic therapy		
No chemo	Referent	
Single‐agent chemo		
At 1 year	1.62 (0.97–2.71)	0.064
At 3 year	1.37 (0.83–2.28)	0.22
At 5 year	1.16 (0.69–1.95)	0.57
Multi‐agent chemo		
At 1 year	1.71 (1.20–2.43)	0.003*
At 3 year	1.44 (1.06–1.97)	0.022*
At 5 year	1.22 (0.91–1.64)	0.19

* Significance of *p*‐value < 0.05.

^a^ The effect estimates for 1‐, 3‐, and 5‐years following diagnosis were presented due to inconsistent association over time (interaction with time) between overall survival and tumor characteristics, patient characteristics, and treatment approaches.

### 3.4 Survival outcomes and treatment approaches for patients with localized/locally advanced disease versus metastatic disease

In the subgroup of patients diagnosed with localized or locally advanced disease and those who underwent either PC or RC (Table 4), higher clinical T‐stage was associated with an increased risk of all‐cause mortality (HR 2.2; 95% CI 1.05–4.69; *p* = 0.04), as compared to clinical T‐stage 2. Receipt of neoadjuvant (HR 1.07; 95% CI 0.10–11.21, *p* = 0.955) or adjuvant (HR 2.28; 95% CI 0.82–6.37, *p* = 0.116) chemotherapy was not associated with OS in this cohort. We found no association between receipt of any chemotherapy and survival (HR 1.97, 95% CI 0.79–4.90, *p* = 0.15). Surgical technique (PC vs. RC) was not associated with OS (HR 1.75, 95% CI 0.72–4.27, *p* = 0.22). A positive surgical margin was not associated with an increased risk of all‐cause mortality (HR 0.81; 95% CI 0.32–2.06, *p* = 0.66).

**TABLE 4:** Multivariable Cox proportional hazards model of overall survival for patients with localized and locally advanced disease who underwent partial or radical cystectomy (cT2‐4, cN0‐1, cM0, *n* = 120)^a^ ^,^ ^b^

	HR (95% CI)	*p*‐value
Age	1.02 (0.99–1.06)	0.16
Insurance status		
No insurance	Referent	
Private	0.01 (0.001–0.04)	<0.001*
Governmental	0.01 (0.002–0.11)	<0.001*
Unknown	0.02 (0.001–0.39)	0.01*
Charlson Deyo Score		
0	Referent	
1	2.43 (0.87–6.79)	0.09
2 and 3	0.98 (0.13–7.26)	0.98
Tumor grade		
Well and moderately	Referent	
Poorly and undifferentiated	0.99 (0.42–2.36)	0.99
Histology		
Non‐mucinous adenocarcinoma	Referent	
Mucinous adenocarcinoma	1.18 (0.54–2.56)	0.68
Signet ring cell carcinoma	1.32 (0.33–5.29)	0.69
Urothelial carcinoma	2.30 (0.26–20.35)	0.46
Squamous cell carcinoma	3.30 (0.40–27.05)	0.27
Surgery type		
Partial cystectomy	Referent	
Radical cystectomy	1.75 (0.72–4.27)	0.22
Clinical T‐stage		
T2	Referent	
T3	2.22 (1.05–4.69)	0.04*
T4	0.57 (0.16–1.97)	0.37
Tumor size		
≤45 mm	Referent	
>45 mm	1.38 (0.61–3.10)	0.44
Surgical margin status		
No residual tumor	Referent	
Positive margin	0.81 (0.32–2.06)	0.66
Receipt of systemic therapy		
No	Referent	
Yes	1.97 (0.79–4.90)	0.15
Regional lymph node dissection		
No	Referent	
Yes	13.13 (4.29–40.20)	<0.001*

* Significance of *p*‐value < 0.05.

^a^ Possible variables for multivariable model were identified through univariable model, literature review, and experts' experience. Significant predictors with *p*‐value <0.05 from univariable analyses and well recognized predictors for survival were included for the final multivariable models.

^b^ Patients with cN1 disease were excluded from the analysis due to insufficient sample size (*n* = 3).

Lastly, in the subgroup of patients diagnosed with metastatic disease, in whom the preferred treatment is systemic therapy, we observed no association between the receipt of chemotherapy and OS (HR 0.785, 95% CI: 0.37–1.65, *p* = 0.52, Table 5). We found that there was no heterogeneity in effect when we examined patients receiving single‐agent or multi‐agent chemotherapy (data not shown), although the sample size in these subgroups was limited. Additionally, we found no heterogeneity of effect for the association between receipt of chemotherapy and survival when stratified by histologic subtypes (mucinous vs. non‐mucinous adenocarcinoma, data not shown).

**TABLE 5:** Hazards ratio (HR) and 95% CI from multivariable Cox proportional hazards model of overall survival for patients with metastatic disease (Stage IV at diagnosis, *n* = 91)^a^

	HR (95% CI)	*p*‐value
Age at diagnosis	1.011 (0.99–1.03)	0.26
Annual income		
Less than $40,227	Referent	
$40,227–$50,353	0.252 (0.096–0.66)	0.005*
$50,354–$63,332	0.529 (0.24–1.18)	0.12
$63,333+	0.173 (0.074–0.41)	0.00*
Insurance		
Not insured	Referent	
Private	0.399 (0.13–1.23)	0.11
Governmental	1.002 (0.33–3.06)	0.997
Unknown	0.072 (0.005–1.06)	0.06
Charlson Deyo score		
0	Referent	
1	1.264 (0.65–2.47)	0.49
2 and 3	0.374 (0.12–1.21)	0.10
Grade		
Well and moderately	Referent	
Poorly and undifferentiated	1.518 (0.59–3.89)	0.38
Missing	0.600 (0.22–1.63)	0.32
Histology		
Non‐mucinous adenocarcinoma	Referent	
Mucinous adenocarcinoma	1.238 (0.59–2.58)	0.57
Signet ring cell carcinoma	18.928 (3.88–92.39)	0.00*
Missing	2.147 (1.01–4.55)	0.05
Clinical N stage		
cN−	Referent	
cN+	1.180 (0.54–2.57)	0.68
cX	1.418 (0.71–2.82)	0.32
Surgery type		
No	Referent	
Local	0.949 (0.44–2.05)	0.89
Partial cystectomy	0.310 (0.13–0.76)	0.01*
Radical cystectomy	0.286 (0.074–1.10)	0.07*
Tumor size		
≤45 mm	Referent	
>45 mm	1.421 (0.66–3.05)	0.36
Missing	1.081 (0.51–2.27)	0.84
Receipt of chemotherapy		
No	Referent	
Yes	0.785 (0.37–1.65)	0.52

* Significance of *p*‐value < 0.05.

^a^ Possible variables for multivariable model were identified through univariable model, literature review, and experts' experience. Significant predictors with *p*‐value <0.05 from univariable analyses and well recognized predictors for survival were included for the final multivariable models.

## 4 DISCUSSION

Due to its rarity, urachal carcinoma has been studied largely through small, single‐ institution series and limited population‐based studies (largest involving 420 patients).^2^, ^4^, ^16^, ^17^ This study thus represents the largest cohort ever used to assess the clinicopathological features and survival outcomes of UCB. We found improved survival with glandular as opposed to non‐glandular histology. In order to evaluate optimal management of these cancers, we assessed outcomes in two cohorts. In patients with localized and locally advanced urachal cancers, LND was performed with increasing utilization for both PC and RC over 2004–2016. In this population, surgical modality (PC vs. RC), margin status, node dissection, and receipt of chemotherapy were not associated with a statistically significant difference in OS. In patients with metastatic disease, which portended a poor survival outcome in this cohort (5‐year OS of 14%, 95% CI 6–24), receipt of systemic chemotherapy was not associated with an improvement in OS.

The current literature on urachal tumors histology (adenocarcinoma) is that the prognosis of either glandular or non‐glandular subtypes are both equally poor.^18^, ^19^ However, our results showed that patients with glandular tumors have better prognosis than those with non‐glandular histology, a novel finding.^4^, ^9^, ^16^ This may be explained by non‐glandular tumors harboring more aggressive histologic features (e.g., mitoses, atypia, or necrosis).

The current recommended treatment for localized disease includes excision of the urachus and bilateral medial umbilical ligaments with PC or RC.^9^, ^10^, ^20^ Previous studies have shown that survival rates are comparable between either surgical modality and our findings were consistent with this.^5^ Patients undergoing PC were, however, less likely to receive regional LND (65.6% vs. 95.2%, *p* = 0.004), while those undergoing RC were more likely to harbor non‐glandular pathology which was shown to portend a poor outcome. The decision to undergo surgery is multifactorial, and requires assessment of patient preferences, bladder functionality (e.g., capacity), and tumor factors, including resectability and location. Ultimately, this may inevitably lead to selection bias for treatment approach.

The role of LND is pivotal in defining cancer staging (pT), as evident in Sheldon, Mayo, and TNM staging guidelines.^5^, ^21^, ^22^ Positive lymph node status is associated with poor prognosis.^10^, ^16^, ^17^ However, the therapeutic role of regional LND in UCB remains controversial in the current literature, though it may provide a prognostic value.^7^, ^12^, ^20^ Szarvas et al. reported lymph node positivity rate of 17% in their cohort of locally advanced or metastatic disease,^9^ which is comparable to that seen in this study (19.1%). We also found that, in both localized and locally advanced disease, patients who underwent LND (*p* < 0.001) had an increased risk in all‐cause mortality, which likely represents selection bias, as those patients harboring worse disease likely preferentially received LND.

Chemotherapy is typically considered for patients with adenocarcinoma of the urachus per NCCN guidelines in the metastatic and adjuvant setting in those with high‐risk pathology, including locally advanced or regionally positive nodal disease.^23^, ^24^ Indeed, in a subset analysis of 74 patients, cisplatin‐based chemotherapy in combination with 5‐FU resulted in improved radiographic progression‐free survival for UC.^9^ The poor outcomes in patients receiving chemotherapy in our entire cohort may thus represent unmeasured bias in patient selection or other factors not accounted for in data. That is, patients destined for worse outcomes are receiving chemotherapy. Interestingly, in the subset of patients with localized/locally advanced disease that were examined, receipt of any systemic chemotherapy was not associated with survival benefit. Other groups using cisplatin‐based chemotherapy and Fluorouracil have similarly found either a lack of demonstrable or limited survival benefit in patients receiving systemic chemotherapy.^3^, ^9^ On the contrary, Flammia et al. showed a significant OS benefit of receiving chemotherapy (16 vs. 3 months) for patients with metastatic UCB, using the Surveillance, Epidemiology, and End Results (SEER) database.^25^ This study used propensity score matching and adjusted for age, sex, race, cystectomy status, and socioeconomic status. Furthermore, this study utilized ICD‐O histology code 8010 or ICD site code C67.7 to build their cohort, which included all the histologies of glandular and non‐glandular subtypes. Thus, the observed differences in survival could be explained by differences in tumor histology or grade, patient comorbidities, or performance status, which are not measured in this dataset and therefore were not adjusted for. Despite these controversial data, the current clinical management of metastatic urachal carcinoma centers on systemic therapy.^24^ There exists a need to design prospective clinical trials to evaluate the most effective regimen and generate data which provide higher level of evidence.

There are several limitations to this study. First, our findings should be interpreted within the retrospective framework of the study. There is a possibility of selection bias, either due to physician practice patterns or patient preferences. Second, data regarding surgical technique, anatomic dissection limits are not reported in NCDB. The mainstay of the treatment, en‐bloc resection of the median umbilical ligament with the umbilicus during the PC, was not recorded by NCDB. Third, the NCDB does not capture several important chemotherapy characteristics, including the type of regimen, the number of cycles, and data regarding response to treatment, making specific practice recommendations impossible. Fourth, our findings can only be generalized to facilities participating in the NCDB. While this includes roughly 70% of all new cancer cases, it may represent a biased sample of facilities with a high volume of oncology referrals and patients. We also found that approximately ~40% patients had an overall unknown AJCC stage, which may reflect a lack of a standardized staging system for urachal carcinoma. The most two common staging systems used are Sheldon and Mayo staging;^5^, ^20^, ^26^ however, the NCDB utilizes the AJCC TNM staging system for bladder, possibly limiting its applicability and leading to noncoding in the NCDB database. Finally, there are no available data to measure cancer‐specific survival. Despite these limitations, our sample size—the largest yet assembled—is the fundamental strength of this analysis of a disease that still lacks study rigor by any prospective randomized clinical trials.

## 5 CONCLUSION

We present the largest cohort ever assembled of patients with this rare disease. Our results implicate that either PC or RC for the management of UCB in localized and locally advanced disease is reasonable, and the decision should be made based on patient preference, comorbidities, and bladder function status. Unlike prior studies, we found no association in OS and positive margin status. Our findings demonstrate that LND has been increasingly performed over the last few years for patients undergoing PC. Systemic chemotherapy was not associated with OS in the management of localized or advanced disease, however. Thus, further studies are warranted to develop novel treatment strategies to improve patient outcomes.

## AUTHOR CONTRIBUTIONS

**Furkan Dursun**: Conceptualization, investigation, methodology; writing – original draft; writing – review & editing. **Kelvin Lim:** Writing – review & editing. **Robert S. Svatek:** Writing – review & editing. **Jiaqiong Xu:** Formal analysis, software, writing – review & editing. **Ziad M. El‐Zaatari:** Writing – review & editing. **Evan P. Wenker:** Writing – review & editing. **Zachary W. Klaassen:** Writing – review & editing. **Ahmed M. Mansour:** Writing – review & editing. **Taliah Muhammad:** Writing – review & editing. **Eleni Efstathiou:** Writing – review & editing. **Guru P. Sonpavde:** Writing – review & editing. **Christopher J.D. Wallis:** Methodology; writing – review & editing. **Raj Satkunasivam:** Conceptualization, investigation, methodology; supervision; data curation; writing – original draft; writing – review & editing.

## CONFLICT OF INTEREST

There are no relevant conflict of interest for all authors.

## ETHICS STATEMENT

This study was exempt from required ethics board approval at our institutions.

## Supporting information


Table S1.

## Data Availability

The primary dataset (National Cancer Database) is available publicly through the American College of Surgeons (https://www.facs.org/quality‐programs/cancer/ncdb). The datasets generated and/or analyzed during the current study are available from the corresponding author upon reasonable request.
