# Supplementary material for: Clinical outcomes and patterns of population‐based management of urachal carcinoma of the bladder: An analysis of the National Cancer Database
Source: Cancer Med. 2022 May 4;11(22):4273–82. doi: 10.1002/cam4.4786 (PMC9678087; doi:10.1002/cam4.4786)
Supplement: Supplementary file 1 — Table S1. [file CAM4-11-4273-s001.docx]

**Supplementary Materials**

**Supplementary Table 1.** AJCC disease stage and grade distribution stratified by major histologic subtype

|  | Non-mucinous Adenocarcinoma  n (%) | Mucinous Adenocarcinoma  n (%) | Signet Ring Cell Carcinoma  n (%) | Urothelial Carcinoma  n (%) | Squamous Cell Carcinoma  n (%) |
| --- | --- | --- | --- | --- | --- |
| **Stage** |  |  |  |  |  |
| Stage 1 | 45 (13.5) | 31 (9.4) | 6 (12.5) | 42 (48.8) | 0 (0) |
| Stage 2 | 55 (16.5) | 43 (13.1) | 6 (12.5) | 0 (0) | 2 (15.4) |
| Stage 3 | 34 (10.2) | 47 (14.3) | 8 (16.7) | 14 (16.3) | 0 (0) |
| Stage 4 | 66 (19.8) | 58 (17.6) | 12 (25) | 12 (13.9) | 7 (53.8) |
| Unknown | 133 (39.9) | 150 (45.6) | 16 (33.3) | 18 (20.9) | 4 (30.8) |
| **Grade** |  |  |  |  |  |
| Well differentiated | 29 (8.7) | 67 (20.4) | 2 (4.2) | 11 (12.8) | 0 (0) |
| Moderate differentiated | 134 (40.2) | 109 (33.1) | 3 (6.2) | 12 (13.9) | 4 (30.8) |
| Poorly differentiated | 79 (23.7) | 46 (14) | 30 (62.5) | 20 (23.3) | 3 (23.1) |
| Undifferentiated, anaplastic | 18 (5.4) | 9 (2.7) | 2 (4.2) | 24 (27.9) | 3 (23.1) |
| Unknown | 73 (21.9) | 98 (29.8) | 11 (22.9) | 19 (22.1) | 3 (23.1) |

**Supplementary Table 2**. Demographic, clinical, and histopathologic characteristics of patients who underwent partial or radical cystectomy for localized and locally advanced disease (cT2-4, cN0-1, cM0)

|  | **Total** | **Partial Cystectomy** | **Radical Cystectomy** | **P-value** |
| --- | --- | --- | --- | --- |
|  | **N=139** | **N=118** | **N=21** |  |
| **Age at Diagnosis** | 55.41±15.05 | 54.40±15.38 | 61.19±11.69 | 0.056 |
| **Sex** |  |  |  | 0.24 |
| Male | 76 (53.52) | 62 (51.24) | 14 (66.67) |  |
| Female | 66 (46.48) | 59 (48.76) | 7 (33.33) |  |
| **Race** |  |  |  | 0.55 |
| White | 110 (77.46) | 93 (76.86) | 17 (80.95) |  |
| Black | 10 (7.04) | 8 (6.61) | 2 (9.52) |  |
| Hispanic | 9 (6.34) | 7 (5.79) | 2 (9.52) |  |
| Asian | 2 (1.41) | 2 (1.65) | 0 (0.00) |  |
| Other/Unknown | 11 (7.75) | 11 (9.09) | 0 (0.00) |  |
| **Insurance Status** |  |  |  | 0.26 |
| Not Insured | 4 (2.82) | 3 (2.48) | 1 (4.76) |  |
| Private | 87 (61.27) | 77 (63.64) | 10 (47.62) |  |
| Governmental | 48 (33.80) | 39 (32.23) | 9 (42.86) |  |
| Unknown | 3 (2.11) | 2 (1.65) | 1 (4.76) |  |
| **Area of Residence** |  |  |  | 1.00 |
| Metro | 96 (72.73) | 80 (72.07) | 16 (76.19) |  |
| Urban | 33 (25.00) | 28 (25.23) | 5 (23.81) |  |
| Rural | 3 (2.27) | 3 (2.70) | 0 (0.00) |  |
| **Annual Income** |  |  |  | 0.62 |
| < $40,227 | 26 (18.31) | 23 (19.01) | 3 (14.29) |  |
| $40,227-50,353 | 36 (25.35) | 30 (24.79) | 6 (28.57) |  |
| $50,354-63,332 | 40 (28.17) | 36 (29.75) | 4 (19.05) |  |
| ≥$63,333 | 40 (28.17) | 32 (26.45) | 8 (38.10) |  |
| **Charlson/Deyo Score** |  |  |  | 0.40 |
| 0 | 107 (75.35) | 91 (75.21) | 16 (76.19) |  |
| 1 | 28 (19.72) | 25 (20.66) | 3 (14.29) |  |
| 2 & 3 | 7 (4.93) | 5 (4.13) | 2 (9.52) |  |
| **Facility Type** |  |  |  | 0.85 |
| Community Cancer Program | 8 (6.67) | 6 (6.00) | 2 (10.00) |  |
| Comprehensive Community  Cancer Program | 47 (39.17) | 40 (40.00) | 7 (35.00) |  |
| Academic/Research Program | 49 (40.83) | 40 (40.00) | 9 (45.00) |  |
| Integrated Network Cancer  Program | 16 (13.33) | 14 (14.00) | 2 (10.00) |  |
| **Clinical T Stage** |  |  |  | 0.17 |
| T2 | 65 (65.66) | 52 (61.90) | 13 (86.67) |  |
| T3 | 32 (32.32) | 30 (35.71) | 2 (13.33) |  |
| T4 | 2 (2.02) | 2 (2.38) | 0 (0.00) |  |
| **Surgical Margin Status** |  |  |  | 0.79 |
| No residual tumor | 117 (82.39) | 99 (81.82) | 18 (85.71) |  |
| Positive surgical margin | 19 (13.38) | 16 (13.22) | 3 (14.29) |  |
| Unknown/others | 6 (4.23) | 6 (4.96) | 0 (0.00) |  |
| **Histology type** |  |  |  | 0.012 |
| Non-mucinous Adenocarcinoma | 66 (46.48) | 58 (47.93) | 8 (38.10) |  |
| Mucinous Adenocarcinoma | 61 (42.96) | 53 (43.80) | 8 (38.10) |  |
| Signet Ring Cell Carcinoma | 8 (5.63) | 7 (5.79) | 1 (4.76) |  |
| UC | 3 (2.11) | 0 (0.00) | 3 (14.29) |  |
| SCC | 4 (2.82) | 3 (2.48) | 1 (4.76) |  |
| **Tumor Grade** |  |  |  | 0.18 |
| Well differentiated | 18 (12.68) | 17 (14.05) | 1 (4.76) |  |
| Moderately differentiated | 58 (40.85) | 49 (40.50) | 9 (42.86) |  |
| Poorly differentiated | 35 (24.65) | 30 (24.79) | 5 (23.81) |  |
| Undifferentiated; anaplastic | 14 (9.86) | 9 (7.44) | 5 (23.81) |  |
| Unknown | 17 (11.97) | 16 (13.22) | 1 (4.76) |  |
| **Tumor size** |  |  |  | 1.00 |
| ≤45 mm | 60 (54.55) | 51 (54.26) | 9 (56.25) |  |
| >45 mm | 50 (45.45) | 43 (45.74) | 7 (43.75) |  |
| **Receipt of Systemic Therapy** |  |  |  | 0.81 |
| No | 105 (73.94) | 89 (73.55) | 16 (76.19) |  |
| Yes | 32 (22.54) | 28 (23.14) | 4 (19.05) |  |
| Unknown | 5 (3.52) | 4 (3.31) | 1 (4.76) |  |
| **Radiation Therapy** |  |  |  | 1.00 |
| No | 130 (91.55) | 110 (90.91) | 20 (95.24) |  |
| Yes | 12 (8.45) | 11 (9.09) | 1 (4.76) |  |
| **Regional Lymph Node Surgery** |  |  |  | 0.004 |
| No | 43 (30.28) | 42 (34.71) | 1 (4.76) |  |
| Yes | 99 (69.72) | 79 (65.29) | 20 (95.24) |  |
